# Effects of Transcranial Neuromodulation on Rehabilitation Outcomes After Anterior Cruciate Ligament Injury: A Systematic Review of Randomized Controlled Trials

**DOI:** 10.3390/biomedicines13123068

**Published:** 2025-12-12

**Authors:** Juan Vicente-Mampel, Mariola Belda-Antolí, Eloy Jaenada-Carrilero, Andrés Pascual-Leone, Luís Baraja-Vegas, Nicolás Pascual-Leone, Javier Ferrer-Torregrosa, Francisco J. Falaguera-Vera, Álvaro Pascual-Leone, José María Tormos-Muñoz

**Affiliations:** 1 School of Medicine and Health Science, Department of Physiotherapy, Catholic University of Valencia, 46001 Valencia, Spain; juan.vicente@ucv.es (J.V.-M.); mariola.belda@ucv.es (M.B.-A.); eloy.jaenada@ucv.es (E.J.-C.); luis.baraja@ucv.es (L.B.-V.); fj.falaguera@ucv.es (F.J.F.-V.); 2 Department of Neurosurgery, Columbia University, New York, NY 10027, USA; apleone97@gmail.com; 3 Hospital for Special Surgery, New York, NY 10021, USA; npascualleone16@gmail.com; 4 School of Medicine and Health Science, Department of Podiatry, Catholic University of Valencia, 46001 Valencia, Spain; 5 Marcus Institute for Aging Research and Wolk Center for Memory Health, Hebrew SeniorLife, Boston, MA 02131, USA; apleone@hsl.harvard.edu; 6 Department of Neurology, Harvard Medical School, Boston, MA 02115, USA; 7 School of Medicine and Health Science, Department of Medicine, Catholic University of Valencia, 46001 Valencia, Spain; jm.tormos@ucv.es

**Keywords:** ACL injury, central nervous system, cortical excitability, muscle strength, neuroplasticity, neuromodulation, tDCS

## Abstract

**Background/Objectives**: Anterior cruciate ligament (ACL) injuries frequently lead to long-term quadriceps impairments despite surgical repair. There is growing evidence that these deficits are caused in part by alterations in the central nervous system. Thus, transcranial neuromodulation (TNM) could be valuable in ACL rehabilitation. To systematically review randomized controlled trials (RCTs) assessing the effects of TNM on neurophysiological, functional, and safety outcomes in patients with ACL injury or reconstruction. **Methods**: We conducted searches on PubMed, Scopus, Web of Science, and Cochrane. We considered all original studies evaluating TNM, including transcranial current stimulation (tCS) and transcranial magnetic stimulation (TMS), in patients with ACL reconstruction or injury. Measures of corticospinal excitability, safety, balance, and muscle strength were assessed. We employed the Cochrane RoB 2 method to assess the risk of bias. **Results:** Seven studies comprising 129 participants (64 TNM, 65 controls) were included. Most studies applied transcranial direct current stimulation (tDCS) over the primary motor cortex contralateral to the ACL injury in conjunction with physical rehabilitation. Single-session protocols demonstrated minimal effects, whereas repeated sessions resulted in improvements in corticospinal excitability, quadriceps strength, and balance. No serious adverse events were reported; minor effects included transient headache or scalp tingling. The risk of bias was assessed as low to moderate across the studies. **Conclusions**: TNM appears to be safe and may enhance functional recovery in individuals with ACL injuries when administered in multiple sessions alongside standard rehabilitation. Further high-quality trials are necessary to determine optimal protocols and long-term outcomes.

## 1. Introduction

Anterior cruciate ligament (ACL) injuries are among the most frequent sports-related knee injuries [1,2], with an incidence of 7 cases per 10,000 people each year [3]. These injuries predominantly affect young and active individuals aged 20–35 years, with 65–75% of tears occurring during athletic activities such as soccer, handball, basketball, and skiing [4,5,6]. Nonetheless, 25–35% of ACL injuries also arise in non-athletic contexts [7]. Males constitute 58–73% of ACL injuries [8,9], although females are 4–8 times more likely to experience an ACL tear when exposure is considered [10,11]. An estimated 70% of affected athletes face a decline in their performance after an ACL tear [1]. ACL reconstruction (ACLR) is the main treatment option for ACL tears, with more than 75% of ACL tears undergoing surgery [6]. However, dynamic and functional instability may persist even after surgical reconstruction [1]. Some patients have unfavorable outcomes after ACLR [4]. Approximately 65% of athletes return to their pre-injury performance level, with even fewer (≈55%) resuming competitive-level activity [12,13]. Quadriceps muscle dysfunction is common and may persist for a prolonged time after ACLR, which can limit functional recovery [5,14].

From a biomechanical standpoint, leg muscles are crucial for stabilizing the knee post-injury. The hamstrings and hip abductors are particularly vital for reducing ACL strain and preventing re-injury, whereas excessive activation of the quadriceps and gastrocnemius may increase anterior tibial translation and ACL stress [15,16]. There is increasing evidence that ACL tears lead to significant alterations in the central nervous system (CNS) [14]. Corticospinal tract (CST) excitability is reduced following ACL tears, limiting motor control and lower extremity function [17]. Post-ACL injury, cortical reorganization occurs within the primary motor cortex, particularly in corticomotor pathways associated with the quadriceps [18,19,20]. These neuroplastic changes, which include altered intracortical and corticospinal excitabilities, contribute to impaired voluntary muscle activation and motor output [21,22]. CNS excitability alterations may contribute to quadriceps dysfunction following ACLR [23].

Traditionally, rehabilitation has concentrated on peripheral recovery; however, conventional physiotherapy methods—such as electrostimulation or isolated strengthening—demonstrate limited effectiveness in addressing central neurophysiological deficits [22]. Recent evidence underscores the significance of integrating neuromodulatory interventions that target both muscular and cortical levels to enhance recovery [24,25,26]. In certain patients, traditional rehabilitation approaches fail to restore quadriceps function fully [27], potentially due to a lack of restoration of normal CST neurophysiology. Therefore, there is growing interest in using interventions that modulate CST excitability in the rehabilitation after ACL injuries [1]. Transcranial neuromodulation (TNM) offers non-invasive and safe means to modulate CST excitability, for example, using transcranial current stimulation (tCS) or transcranial magnetic stimulation (TMS) [28,29]. TNM targeting the primary motor cortex (M1) can increase CST excitability and has been shown to improve functional recovery in targeted muscles [30,31].

A recent meta-analysis of 44 RCTs with 1555 post-stroke patients demonstrated that transcranial direct current stimulation (tDCS) improved upper extremity motor function (standardized mean difference = 0.22, 95% confidence interval: 0.12–0.32, *p* < 0.001) [32]. Krogh et al. [33] reported that 4 weeks of repetitive TMS combined with resistance training significantly improved Lower Extremity Motor Score (LEMS) in individuals with spinal cord injury compared to sham control. Given that motor cortex excitability may be reduced in ACLR patients, TNM may theoretically improve recovery by increasing CST activation during exercise [4,14]. However, while TNM has been shown to improve clinical outcomes in various neurological and musculoskeletal disorders [33,34,35,36], its efficacy in ACL injuries remains unclear. We aim to assess the existing evidence on the impact of TNM on ACL injuries. Our research question is articulated using the PICO framework as follows: In patients with anterior cruciate ligament injury or reconstruction (P), does transcranial neuromodulation (I), in comparison to usual care or sham stimulation (C), enhance neurophysiological, functional, and safety outcomes (O)? This inquiry directs the systematic evaluation of existing randomized controlled trials in this domain.

## 2. Materials and Methods

### 2.1. Protocol Registration

We conducted this review in accordance with the Preferred Reporting Items for Systematic Reviews and Meta-Analyses (PRISMA) [37] guidelines and the Cochrane Handbook for Systematic Reviews of Interventions [38]. The systematic review process, including independent screening, data extraction, quality assessment, and synthesis, was conducted from 25 June to 25 July 2025, adhering to standard systematic review timelines for methodological rigor, and the protocol was registered in PROSPERO on 1 July 2025 under the ID: CRD420251080478.

### 2.2. Data Sources and Search Strategy

Four databases (PubMed, Scopus, Web of Science, and Cochrane) were searched from inception until April 2025, using the following keywords: “Transcranial Neuromodulation”, “Transcranial Electrical Stimulation”, “Transcranial Magnetic Stimulation”, and “Anterior Cruciate Ligament Reconstruction”. The detailed search strategy is illustrated in File S1. The systematic review process, including independent screening, data extraction, quality assessment, and synthesis, was conducted from June to July 2025, adhering to standard systematic review timelines for methodological rigor.

### 2.3. Inclusion Criteria

The inclusion criteria were established using the PICO framework. We included any original study that assessed TNM on patients with ACL injury or after ACLR. Patients diagnosed with ACL injury or after ACLR comprised the population (P). Intervention (I): TNM regardless of modality (e.g., electrical, magnetic, ultrasound, or light-based) or stimulation frequency, while comparison (C) involved control groups receiving usual care or sham stimulation. Primary outcomes (O) of interest included neurophysiological, functional, and safety endpoints. We excluded studies that used peripheral stimulation, single-arm non-controlled studies, reviews, editorials, and animal studies. No restrictions were applied regarding language or publication year.

### 2.4. Study Selection

We imported the identified records into EndNote 2025 (Clarivate Analytics, Philadelphia, PA, USA) to remove duplicates. Two independent authors screened the title and abstract, followed by full-text screening of the relevant studies using the Rayyan website [39]. Moreover, we performed forward and backward citation analyses for the included studies. Any conflict between the authors was addressed through consensus.

### 2.5. Data Extraction

Two independent authors extracted the data using a predefined Excel sheet. The extracted data was the study summary, including study ID, study design, country, sample size for each group, ACL condition, type of TNM used, type of comparator, target brain region, concurrent treatments, follow-up duration, endpoints in each study, and conclusion. Baseline characteristics included: age, male sex, height, weight, and body mass index (BMI). Additionally, we completed a risk of bias assessment.

### 2.6. Risk of Bias and Quality Assessment

We employed the ROB2 tool to evaluate the risk of bias [40]. This instrument assessed the randomized clinical trials across five domains: the randomization process, deviations from intended interventions, missing outcome data, outcome measurement bias, and reporting bias. The methodological quality of the included studies was independently evaluated by two reviewers (M.B.-A. and F.F.-V.). In instances of disagreement, a third and fourth reviewer (A.P.-L. and N.P.-L.) were consulted, and the final decision was reached through consensus among all reviewers.

### 2.7. Evidence Synthesis

Because of the variability in intervention regimen, outcome measures, and follow-up lengths, we used a descriptive evidence synthesis. No meta-analysis was performed. Conducting a quantitative synthesis through meta-analysis was not possible because of significant differences among the studies, including variations in species, pain models, types of seaweed, chemicals examined, study designs, and methods of measuring outcomes. Consequently, we opted for a narrative synthesis of the evidence to address this gap. This methodological decision aligns with the Cochrane guidelines, which recommend narrative synthesis when heterogeneity exceeds the appropriate statistical thresholds. We compared the main findings across different models and study contexts, organizing the studies by compound class and the seaweed species. Where applicable, we explored the potential mechanisms of action and examined the patterns of consistency or variability of the included studies. Furthermore, we analyzed stimulation parameter-outcome relationships to identify potentially optimal protocols, recognizing the clinical relevance of protocol optimization for future applications.

## 3. Results

### 3.1. Search Results and Study Selection

**Figure 1:**
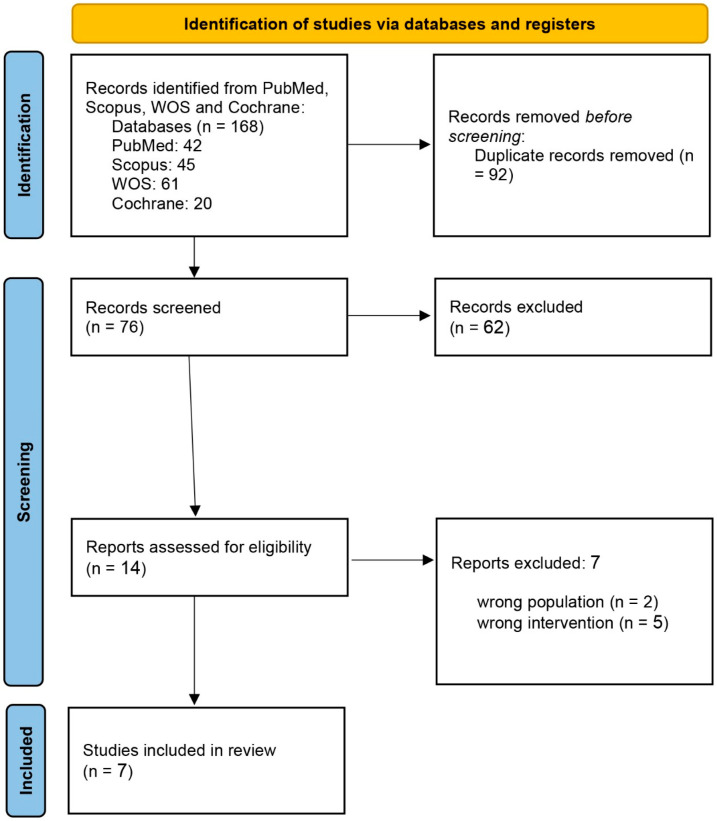
PRISMA flow chart of the screening process.

At the initial systematic search, 168 eligible studies were determined. Of these, 92 articles were duplicated, and another 62 were not qualified based on the information presented in the abstract. Finally, the full texts of 14 studies were reviewed, and 7 were included in the systematic review. The PRISMA flow chart of the selection process is shown in Figure 1.

### 3.2. Study Characteristics

Seven randomized controlled trials, including two cross-over trials [4,27], assessed TNM interventions in individuals with ACL injury or ACL reconstruction. Across all studies, there were a total of 129 participants with parallel-group and crossover designs (sample sizes per study ranged from 6 to 34 individuals).

The participants were predominantly young adults, with a mean age ranging from 20.6 to 34.8 years. Some studies, such as Reuter et al. [1], reported BMI values of 27.1 (SD = 3.6) in TNM and 25.9 (SD = 1.9) in controls. Several studies, such as Jamebozorgi et al. [23], enrolled only male participants. TNM modalities primarily targeted the primary motor cortex (M1), with six studies using tDCS with the anode over the motor cortex (M1) contralateral to the ACL injury (a-tDCS). In these tDCS studies, stimulation levels of 1 to 2.2 mA were applied for 20 min, with protocols varying from single sessions to ten sessions within a six-week timeframe. One study (Jamebozorgi et al., 2023) [23] used tDCS with the anode over Oz (occipital cortex) and the cathode over the right shoulder. Jamebozorgi et al. (2023) [23] aimed to enhance visual-proprioceptive integration to improve balance and proprioception outcomes. Flanagan et al. (2021) [41] utilized intermittent theta burst stimulation (iTBS) with M1 as the target. Follow-up assessments ranged from immediate post-intervention to six weeks. All studies except one (Flanagan et al., 2021) [41] used concurrent treatment as summarized in Table 1.

Table 1, Table 2 and Table 3 provide the summary and main findings, and baseline characteristics of the included studies, respectively.

#### Stimulation Parameters and Clinical Outcomes Analysis

Seven RCTs employed variable stimulation parameters: anodal tDCS intensity ranged from 1 to 2.2 mA, duration was consistently 20 min, and session frequency varied from single sessions to 10 sessions over 4–6 weeks. Six studies targeted the primary motor cortex (M1) contralateral to the ACL injury, while one study (Jamebozorgi et al., 2023) [23] used occipital cortex targeting for visual-proprioceptive integration.

Protocol Effectiveness Patterns: 1-Multi-session protocols (≥3 sessions) demonstrated superior clinical outcomes compared to single sessions; 2-M1 targeting showed consistent improvements in corticospinal excitability and muscle function; 3-Occipital targeting enhanced proprioception and balance measures specifically; 4-Session frequency rather than intensity appeared to be the primary determinant of clinical efficacy.

These findings suggest that protocol design (session number and cortical targeting) may be more critical than stimulation intensity for optimal clinical outcomes in ACL rehabilitation.

### 3.3. Patient Population

The participants in the studies had different stages of ACL pathology, including acute ACL rupture (Murphy et al., 2024 [6]; Reuter et al., 2024 [1]; Tohidirad et al., 2023 [2]), subacute injuries (Tohidirad et al., 2023 [2]), and post-operative ACL reconstruction (Zarzycki et al., 2025 [27]; Rush et al., 2020 [4]). Participants were generally enrolled if they had an ACL injury or underwent ACL reconstruction with adequate knee range of motion, minimal knee effusion, and no active infection. There were consistent exclusion criteria regarding previous knee surgery (aside from the ACL reconstruction), multi-ligament injuries, neuromuscular disorders, and contraindications to TNM (e.g., metal implants, history of seizures) [1,2,4,6,27].

### 3.4. Outcomes

The findings are presented descriptively, given the clinical and methodological heterogeneity across studies, which precludes meaningful statistical pooling. This approach allows for a transparent representation of diverse intervention protocols while maintaining methodological rigor appropriate for this emerging research area.

A broad range of outcomes was assessed across the studies, which can be categorized into three main domains.

Neurophysiological outcomes: Corticospinal excitability and motor cortex function showed variable responses in different studies. Two studies (Zarzycki et al., 2025 [27]; Rush et al., 2020 [4]) reported non-significant changes in active motor thresholds and muscle function following single-session TMS protocols. In contrast, Murphy et al. [6] demonstrated significant improvements in quadriceps intracortical inhibition and facilitation using multi-session tDCS protocols. Table 2 provides the detailed statistical findings for each study.

Functional performance and balance outcomes: Related to postural control, proprioception, and muscle function. Mixed findings were observed for balance and proprioception measurements across studies. Reuter et al. [1] observed non-significant reductions in medial-lateral and anterior–posterior center of pressure (CoP) displacements post-TNM, with no *p*-values reported. The CoP velocity also slightly declined in both groups. In contrast, Tohidirad et al. [2] reported significant improvements in several postural control metrics: CoP displacement in the *y*-axis for both legs increased by 26.68 mm (SD 10.43; *p* = 0.018), and APF at 60 ms rose by 9.23 N (SD 2.20; *p* = 0.001). Proprioception and dynamic balance were assessed using the joint position error and the Star Excursion Balance Test (SEBT), respectively. Jamebozorgi et al. [23] reported greater anterior SEBT improvements in the TNM group (12.4 cm, SD 0.23) than controls (1.5 cm, SD 0.83), but without statistical significance (*p* = 0.3). Knee joint position sense errors showed no significant between-group differences at 30°, 45°, or 90° (all *p* > 0.05). Table 2 provides detailed findings for each of the studies.

Rush et al. [4] evaluated muscle function and EMG activity and found non-significant changes in EMG (% change in vastus medialis: −12.1% vs. −18.9%, *p* = 0.31; vastus lateralis: −14.8% vs. −25.9%, *p* = 0.25), isometric strength (−8.9% vs. −10.1%, *p* = 0.75), and voluntary activation (−5.03% vs. −5.5%, *p* = 0.79). Patient-reported outcomes, including the Knee Injury and Osteoarthritis Outcome Score (KOOS) subscales, showed modest improvements that were not statistically significant. For instance, KOOS pain improved by 2.8 points in the TNM group versus 0.6 points in the control group (*p* = 0.47), and KOOS symptoms improved by 4.7 versus 3.1 points (*p* = 0.09).

Safety outcomes: Adverse events were consistently minor and well tolerated across all studies. Minor symptoms, such as headache, sleepiness, and scalp tingling, were infrequent and resolved without intervention, with no serious adverse events reported. These safety findings are consistent with the established safety profiles of transcranial neuromodulation techniques in clinical populations. Table 2 provides detailed safety data for each of the studies.

Note on evidence visualization: Visual summaries, such as forest plots or direction-of-effect matrices, are most informative when sufficient homogeneous studies are available for statistical pooling. Given the clinical and methodological heterogeneity identified across our included studies (see Table 1, Table 2 and Table 3), such visualizations would not meaningfully contribute to the evidence synthesis and could potentially mislead readers about the certainty of available evidence.

### 3.5. Risk of Bias

**Figure 2:**
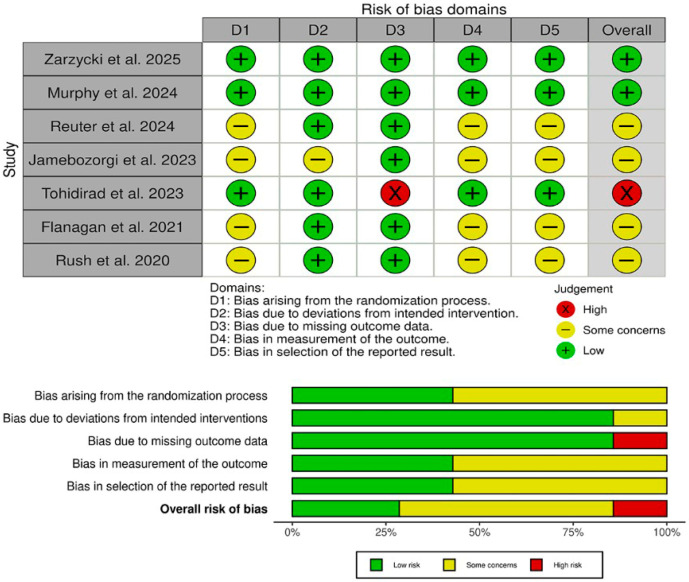
Quality assessment of risk of bias in the included trials. The upper panel presents a schematic representation of risks (low = green, unclear = yellow, and high = red) for specific types of biases of each of the studies in the review. The lower panel presents risks (low = green, unclear = yellow, and high = red) for the subtypes of biases of the combination of studies included in this review [1,2,4,6,23,41].

The risk of bias assessment using the Cochrane RoB 2 tool (Figure 2) revealed that two studies (Zarzycki et al. [27] and Murphy et al. [6]) achieved an overall low risk of bias, with all five domains (randomization, deviations from intended intervention, missing data, outcome measurement, and selective reporting) judged as low risk. One trial (Tohidirad et al. [2]) was rated high overall due to inadequate handling of missing outcome data (D3), despite low risk in other domains. The remaining four studies (Reuter et al. [1], Jamebozorgi et al. [23], Flanagan et al. [41], and Rush et al. [4]) each had “some concerns” overall, most often reflecting unclear allocation procedures (D1), incomplete blinding of outcome assessors (D4), or potential selective reporting (D5). These findings underscore that, while the two well-controlled RCTs provide the most reliable evidence, results from smaller pilot and quasi-experimental studies should be interpreted with caution.

## 4. Discussion

This systematic review evaluates synthesized evidence from seven controlled trials examining TNM in ACL-injured populations. Our findings suggest TNM, especially paired with rehabilitation, might offer promise for enhancing functional outcomes. All, but one of the studies included in the review were conducted with tDCS, and in most cases the anode was placed over M1 contralateral to the injured ACL. Although single session tDCS protocols did not elicit changes in CST excitability or muscle contraction (i.e., strength), reductions in quadriceps inhibition [6,27] and improvements in dynamic stability [2] were observed after several sessions. This finding is consistent with the literature on noninvasive brain stimulation in other rehabilitation applications, which emphasizes the value of combination with physical, occupational, or speech therapy interventions, and highlights the need for multiple intervention sessions to achieve meaningful clinical outcomes. The single study with iTBS [41], points to the possibility of longer-lasting changes to brain organization and function, but the focus on injury phases suggests more work is needed to determine optimal timings and sequences for intervention with neuromodulation.

Cross-study comparisons are difficult owing to different co-interventions and protocols. Nonetheless, the findings support the value of integrating noninvasive brain stimulation techniques [42], particularly tDCS, into rehabilitation programs to improve motor recovery after ACL reconstruction surgery [30]. However, larger, well-controlled studies are needed, and the mechanisms of action remain unclear. For instance, Murphy et al. (2024) [6] found that multi-session tDCS with the anode applied to M1 during rehabilitation promotes suppression of quadriceps motor cortex intracortical inhibition and enhancement of intracortical facilitation. This alleviates the effects of AMI, which is a very common ACL injury complication and has a detrimental effect on neuromuscular function [4]. However, Jamebozorgi et al. [23] who combined tDCS with biofeedback found improved proprioception leading to the improved balance in athletes with ACL deficiency [23]. It is likely that TNM in ACL rehabilitation may have therapeutic effects related to different neurophysiological pathways. TNM may modulate and normalize CST excitability, reduce AMI, restore the balance of interhemispheric inhibition. improve dynamic balance by modulating hyperactive reflex arcs at the spinal level and strengthen sensorimotor integration and support the Hebbian plasticity hypothesis [43]. In any case, TNM combined with physical and other therapies can yield functional gains beyond those achieved with sham stimulation, suggesting that TNM can prime the motor system towards recovery [44].

The neurobiological mechanisms underlying the effects of TNM may involve the modulation of cortical excitability and synaptic plasticity [45]. Specifically, principles of Hebbian plasticity suggest that the repeated co-activation of cortical and muscular pathways can strengthen neural connections, thereby enhancing motor recovery [46]. Modulation of short-interval intracortical inhibition and intracortical facilitation (SICI/ICF) may alleviate AMI by reducing excessive cortical inhibition while promoting excitatory pathways [47]. Improvements in sensorimotor integration and proprioception may result from enhanced communication between sensory and motor cortical regions, thereby supporting coordinated movement and balance [48]. Furthermore, evidence from related interventions underscores the extensive neurophysiological and psychological potential of TNM and complementary practices. For instance, a study employing a single session of tDCS in conjunction with mirror therapy demonstrated a direct enhancement in the function of the dominant hand and an indirect improvement in the non-dominant hand [49]. This indicates that even acute neuromodulatory interventions can augment cortical plasticity and motor function. Additionally, mind–body interventions, such as a 3-month yoga and meditation retreat, have been associated with improvements in psychological well-being, increased BDNF and cortisol awakening response, and modulation of inflammatory markers [50]. These findings suggest that interventions targeting neuroplasticity and physiological regulation can facilitate mind–body integration, potentially complementing TNM in rehabilitation by supporting overall neural and systemic health.

Finding the ideal timing concerning injury or surgical intervention, identifying patient subgroups who respond well, and improving stimulation parameters (such as intensity, duration, and electrode montage) are critical areas. The integration of TNM with rehabilitation warrants further exploration to disentangle synergistic effects from confounding co-interventions. Multimodal assessments, along with neurophysiological (e.g., TMS, EEG), biomechanical, and patient-reported outcomes, are needed to clarify underlying mechanisms and functional relevance. Additionally, emerging technology that includes closed-loop NIBS and individualized dosing based on baseline cortical excitability might also enhance treatment precision and efficacy. Longitudinal studies comparing retention of benefits, re-damage risk, and return-to-sport metrics will be important for translating these neuromodulatory techniques into clinical exercise. NIBS techniques delivered over several sessions may enhance neuromuscular recovery following ACL injuries or reconstructive surgeries.

### 4.1. Limitations

In summary, this systematic review provides the first comprehensive synthesis of randomized controlled trial data on TNM for ACL rehabilitation. Overall, the available studies highlight the potential of TNM, particularly tDCS when paired with physical therapy, to promote ACL injury recovery and improve balance. However, the studies published to date are hindered by their small sample sizes, differing methodologies, and lack of follow-up, which prevents drawing firm conclusions and underscores the necessity for further evidence. Most available studies as single center with varying blinding and control settings, and a higher risk of bias. Furthermore, while the diversity of methods limits the comparability across studies, the complete absence of quantitative analysis diminishes the review’s evidential strength. Although a simple meta-analysis (e.g., standardized mean difference for muscle strength or balance) employing a random-effects model could have been considered, the heterogeneity of protocols and outcomes led us to provide a rationale for the absence of a meta-analysis.

Study quality considerations significantly impacted the evidence in our review. Two studies demonstrated a low risk of bias across all domains, providing high-certainty evidence, particularly for safety outcomes. However, most of the included studies (*n* = 4) showed “some concerns” primarily due to unclear allocation concealment and incomplete outcome assessor blinding, while one study was rated as having a high risk due to inadequate handling of missing data. The small sample sizes across most studies (6–34 participants) limit generalizability and increase uncertainty around effect estimates. These quality concerns suggest that while the TNM appears promising for ACL rehabilitation, the findings should be interpreted with appropriate caution, particularly for subjective functional outcomes. Future high-quality, large-scale trials are essential before definitive clinical recommendations can be formulated.

### 4.2. Implications for Future Research

There is a need for multi-center, large-scale randomized controlled trials with more precise parameters. Ideally, future studies would incorporate large participant pools, rigorous randomization and blinding, set stimulation parameters such as site, intensity, and dosage, and measure clinically relevant outcomes like quadriceps strength, functional performance, comprehensive patient-reported outcomes, and long-term multicenter follow-up. Addressing these limitations would enhance the generalizability of the findings and allow determination of the value of TNM in standard rehabilitation protocols following ACL surgeries.

## Figures and Tables

**Table 1:** Summary characteristics of the included RCTs.

Study ID	Study Design	Country	Total Sample Size		ACL Condition	Inclusion Criteria	Type of TNM	Target Brain Region	Concurrent Treatments	Follow-Up Duration	Endpoints
			TNM	Control							
Zarzycki et al., 2025 [27]	A randomized crossover design	USA	10	10	Anterior Cruciate ligament reconstruction	Participants aged between 18 and 42 years who were 4 to 6 months post-primary anterior cruciate ligament reconstruction (ACLR). All participants had a “quiet knee,” defined as full range of motion, minimal or no effusion, and no observable gait deviation.	Anodal tDCS (single session)	Primary motor cortex (M1)	Stationary cycling	The outcomes were reported immediately. No long-term follow-up	Active Motor Threshold, Slope of the stimulus-response curve, Peak Torque, and Rate of Torque Development
Murphy et al., 2024 [6]	A triple-blind, randomized controlled trial	Australia	11	10	Acute ACL rupture, post-reconstruction	Participants aged 18–60 with a primary, non-contact acute ACL rupture from type 1 or 2 physical activity, requiring surgical reconstruction with an ipsilateral hamstring tendon graft	Anodal tDCS (2 mA, 20 min, three times per week)	Primary Motor cortex (M1)	exercise-based rehabilitation	6 Weeks	Active Motor Threshold, Maximal Voluntary Isometric Contraction, Quadriceps, Pre-TMS EMG (Quadriceps, Hamstrings), Short Intracortical Inhibition, Quadriceps, Long Intracortical Inhibition, Quadriceps, Short Interval Cortical Facilitation, Quadriceps, Hamstrings
Reuter et al., 2024 [1]	a sham-controlled randomized pilot study.	Germany	6	6	a complete unilateral tear of the ACL	Patients with a complete unilateral ACL tear within the past 90 days who underwent arthroscopic reconstruction using an ipsilateral semitendinosus tendon graft were able to fully weight-bear, demonstrated quadriceps control, and had at least 90° knee flexion and full extension	Anodal (2 mA, 20 min, 3 sessions/week)	Primary Motor cortex	Sensorimotor training (balance and stability exercises, 3×/week for 6 weeks)	6 weeks	CoP ML (mediolateral), CoP AP (anteroposterior), CoP Velocity
Jamebozorgi et al., 2023 [23]	quasi-experimental study.	Iran	11	11	isolated and complete ACL rupture	subjects with an isolated and complete ACL injury in the 18–30 age range, injured for at least four months, and diagnosed with an ACL rupture by an orthopedist using MRI	Anodal tDCS (1 mA, 20 min, 10 sessions)	Occipital cortex	Isometric contraction exercises	4 weeks	The Star Excursion Balance Test (SEBT) (Anterior, Lateral, Posterior), Knee absolute error (at 30°, 45°, and 90°)
Tohidirad et al., 2023 [2]	Double-blind, randomized clinical trial	Iran	17	17	Partial ACL rupture	Participants were included if they were aged from 22 to 40 years, had an acute and subacute phase of injury (maximum 1 months after injury), incurred partial rupture of ACL on the right side, had a body mass index of 20 to 25, and had at least 1 year experience in the sport.	Anodal tDCS (2 mA, 20 min, 10 sessions)	primary motor cortex (M1)	different physical therapy (PT) techniques	4 weeks	Center of pressure (COP), Ankle plantar flexor (APF), ankle plantar extension (APE)
Flanagan et al., 2021 [41]	randomized, shame-controlled, double-blind, crossover study design.	USA	9	11	unilateral anterior cruciate ligament rupture	N.A.	intermittent theta burst stimulation (iTBS)	Primary motor cortex (M1)	N.A.	N.A.	T VL EMG, VL: BF coactivation, BF EMG
Rush et al., 2020 [4]	A randomized crossover design	USA	10	10	Anterior Cruciate Ligament Reconstruction	To be included, participants reported the history of a unilateral ACLR and were a minimum of 6 months post-reconstruction with full clearance for return to activity/sport by their physician.	Anodal tDCS (2.2 mA, 20 min, one session)	Primary Motor Cortex (M1)	Walking on a treadmill	Immediate Post- Intervention Assessment. No long-term follow-up	VM% %EMG max Activity, VL% %EMG max Activity, Isometric Strength, central activation ratio (CAR), KOOS Sx, KOOS Pn

Abbreviations: ACL: Anterior Cruciate Ligament, APF: ankle peak force, APE: ankle proprioception error, BF: Biceps Femoris, CAR: central activation ratio, CoP: center of pressure, EMG: Stimulation Electromyography, KOOS: Knee Injury and Osteoarthritis Outcome Score, KOOS Sx: Symptoms subscale of KOOS, KOOS Pn: Pain subscale of KOOS, N.A.: Not Available, Pre-TMS: Pre-Transcranial Magnetic, SEBT: Star Excursion Balance Test, TNM: Transcranial neuromodulation, tDCS: transcranial direct current stimulation, VL: Vastus Lateralis, VM: Vastus Medialis.

**Table 2:** Summary of Main Findings from Recent Studies on tDCS and Neuromuscular Function Following ACL Reconstruction.

Study ID	Main Findings
Zarzycki et al., 2025 [27]	tDCS is safe and feasible. There was no significant condition by time interaction for CSE (*p* ≥ 0.17) or quadriceps performance (*p* ≥ 0.53), though there was a significant main effect of time for RTD200 (*p* = 0.02) with decreased RTD200 post-intervention regardless of condition.
Murphy et al., 2024 [6]	Anodal-tDCS selectively modulated quadriceps excitability, producing significant group-by-time interactions in SICI and SICF, while quadriceps LICI remained unchanged. Quadriceps MVIC significantly increased over time regardless of group (β = 60.667, *p* = 0.004). In the hamstrings, inhibition (SICI) increased over time, LICI consistently differed between groups, and SICF showed a significant group-by-time interaction without overall time or group effects.
Reuter et al., 2024 [1]	Sensorimotor training has a significant effect on CoP ML (*p* = 0.025) and CoP in AP direction (*p* = 0.03) in the leg, where an anterior cruciate ligament tear occurred. but the addition of anodal transcranial direct-current stimulation placed over the primary motor cortex did not potentiate the adaptive responses of the sensorimotor training.
Jamebozorgi et al., 2023 [23]	No significant between-group differences were found for knee proprioception or functional balance (SEBT) across biofeedback, tDCS, and control conditions (*p* > 0.05). Within-group analyses showed that biofeedback improved SEBT performance in all eight directions and proprioceptive accuracy at 30°, 45°, and 90° (*p* < 0.05), while tDCS enhanced balance in six directions (all but anterior-lateral and posterior) and proprioception at all tested angles.
Tohidirad et al., 2023 [2]	One month after treatment, the displacement of the pressure center decreased in the intervention group (*p* < 0.05), while there were no changes in the control group. *Y*-axis of center of pressure decreased in the intervention group relative to the control group, although the average of power of flexor and extensor muscles increased immediately in both groups, but the rise in the intervention group was larger than that in the control group (*p* < 0.05).
Flanagan et al., 2021 [41]	In ACL participants, iTBS significantly increased injured-leg quadriceps activation (↑ T VL EMG, *p* = 0.05) and eliminated asymmetry in quadriceps-hamstring coactivation (VL: BF ratio, *p* = 0.25), normalizing neuromuscular function. Conversely, iTBS reduced quadriceps activation (↓ T VL EMG, *p* = 0.01) and impaired force production in controls.
Rush et al., 2020 [4]	Active tDCS showed no significant benefit over sham stimulation: Both conditions resulted in immediate declines in quadriceps EMG activity, strength, and voluntary activation after exercise, with similarly small (non-significant) improvements in self-reported pain and symptoms. The lack of between-group differences indicates that a single tDCS session does not acutely enhance muscle function or reduce pain in ACL-reconstructed individuals.

Abbreviations: ACL: Anterior Cruciate Ligament, AP: Anteroposterior, BF: Biceps Femoris, CSE: Corticospinal Excitability, CoP: center of pressure, EMG: Stimulation Electromyography, iTBS: Intermittent Theta-Burst Stimulation, LICI: Long-Interval Intracortical Inhibition, ML: Mediolateral, MVIC: Maximal Voluntary Isometric Contraction, RTD: Rate of Torque Development, SEBT: Star Excursion Balance Test, SICF: Short-Interval Intracortical Facilitation, SICI: Short-Interval Intracortical Inhibition, tDCS: transcranial direct current stimulation, VL: Vastus Lateralis. ↑ means increase, ↓ means decrease.

**Table 3:** Baseline characteristics of the participants.

Study ID	Age (Years), Mean (SD)		Male (N)		Height (cm), Mean (SD)		Weight (kg), Mean (SD)		BMI, Mean (SD)	
	TNM	Control	TNM	Control	TNM	Control	TNM	Control	TNM	Control
Zarzycki et al., 2025 [27]	23.9 (6.3)		9		N.A.		N.A.		24.3 (3.3)	
Murphy et al., 2024 [6]	24 (5)	25 (5)	13		173 (8)	181 (12)	77 (15)	86 (18)	N.A.	N.A.
Reuter et al., 2024 [1]	31.8 (4.8)	34.8 (8.4)	5		176.2 (2.5)	166.8 (13.1)	84.2 (11)	72.5 (12.8)	27.1 (3.6)	25.9 (1.9)
Jamebozorgi et al., 2023 [23]	29.91 (8.75)	30.25 (1.25)	All were male.		177 (7.26)	178 (5.35)	81.37 (16.36)	78.75 (5.37)	25.85 (4.49)	24.62 (1.75)
Tohidirad et al., 2023 [2]	31.53 (7.2)	31.73 (7.05)	10	9	169.4 (11.4)	170.73 (11.13)	72.53 (15)	75.86 (20)	24.7 (4.44)	26.15 (5.3)
Flanagan et al., 2021 [41]	20.6 (2.3)	20.3 (1.4)	All were females.		166.1 (8)	165 (5.3)	68.1 (9.1)	65.7 (8.4)	N.A.	N.A.
Rush et al., 2020 [4]	22.9 (4.23)		5		175.26 (10.86)		80.87 (16.86)		N.A.	

Abbreviations: BMI: body mass index, N.A.: Not Available, N: number, SD: standard deviation, TNM: Transcranial neuromodulation.

## Data Availability

No new data were created or analyzed in this study.

## Supplementary Materials

The following supporting information can be downloaded at: https://www.mdpi.com/article/10.3390/biomedicines13123068/s1, Table S1. Search strategy table based on each database. File S1: PRISMA 2020 checklist neuromodulation in acl rehabilitation.
