# Supplementary material for: Effects of Transcranial Neuromodulation on Rehabilitation Outcomes After Anterior Cruciate Ligament Injury: A Systematic Review of Randomized Controlled Trials
Source: Biomedicines. 2025 Dec 12;13(12):3068. doi: 10.3390/biomedicines13123068 (PMC12730971; doi:10.3390/biomedicines13123068)
Supplement: Supplementary file 1 [file biomedicines-13-03068-s001.zip › Table S1.pdf]

Table S1. Search strategy table based on each database.

Database	Search strategy	Search field	Results
**Pubmed**	((("transcranial"[All Fields] OR "transcranially"[All Fields]) AND ("neuromodulate"[All Fields] OR "neuromodulating"[All Fields] OR "neuromodulation"[All Fields] OR "neuromodulations"[All Fields] OR "neuromodulative"[All Fields] OR "neurotransmitter agents"[Pharmacological Action] OR "neurotransmitter agents"[Supplementary Concept] OR "neurotransmitter agents"[All Fields] OR "neuromodulator"[All Fields] OR "neuro-transmitter agents"[MeSH Terms] OR ("neurotransmitter"[All Fields] AND "agents"[All Fields]) OR "neuromodulators"[All Fields])) OR ("Transcranial Direct Current Stimulation"[MeSH Terms] OR ("transcranial"[All Fields] AND "direct"[All Fields] AND "current"[All Fields] AND "stimulation"[All Fields]) OR "Transcranial Direct Current Stimulation"[All Fields]) OR ("Tran-scranial Direct Current Stimulation"[MeSH Terms] OR ("transcra-nial"[All Fields] AND "direct"[All Fields] AND "current"[All Fields] AND "stimulation"[All Fields]) OR "Transcranial Direct Current Stimulation"[All Fields] OR "tdcs"[All Fields]) OR ("tran-scranial magnetic stimulation"[MeSH Terms] OR ("transcra-nial"[All Fields] AND "magnetic"[All Fields] AND "stimula-tion"[All Fields]) OR "transcranial magnetic stimulation"[All Fields]) OR ("Anodal"[All Fields] AND ("stimulate"[All Fields] OR "stimulated"[All Fields] OR "stimulates"[All Fields] OR "stimulat-ing"[All Fields] OR "stimulation"[All Fields] OR "stimulations"[All Fields] OR "stimulative"[All Fields] OR "stimulator"[All Fields] OR "stimulator s"[All Fields] OR "stimulators"[All Fields])) OR ("Cathodal"[All Fields] AND ("stimulate"[All Fields] OR "stimu-lated"[All Fields] OR "stimulates"[All Fields] OR "stimulating"[All Fields] OR "stimulation"[All Fields] OR "stimulations"[All Fields] OR "stimulative"[All Fields] OR "stimulator"[All Fields] OR "stim-ulator s"[All Fields] OR "stimulators"[All Fields])) OR "Transcra-nial Direct Current Stimulation"[MeSH Terms] OR ("Transcranial Direct Current Stimulation"[MeSH Terms] OR ("transcranial"[All Fields] AND "direct"[All Fields] AND "current"[All Fields] AND "stimulation"[All Fields]) OR "Transcranial Direct Current Stimu-lation"[All Fields] OR ("transcranial"[All Fields] AND "electri-cal"[All Fields] AND "stimulation"[All Fields]) OR "transcranial electrical stimulation"[All Fields])) AND ("anterior cruciate liga-ment"[MeSH Terms] OR ("anterior"[All Fields] AND "cruci-ate"[All Fields] AND "ligament"[All Fields]) OR "anterior cruciate ligament"[All Fields] OR ("anterior cruciate ligament"[MeSH Terms] OR ("anterior"[All Fields] AND "cruciate"[All Fields] AND "ligament"[All Fields]) OR "anterior cruciate ligament"[All Fields] OR "acl"[All Fields]) OR (("anterior cruciate ligament"[MeSH Terms] OR ("anterior"[All Fields] AND "cruciate"[All Fields] AND "ligament"[All Fields]) OR "anterior cruciate ligament"[All Fields]) AND "injur*"[All Fields]) OR ("Anterior Cruciate Ligament Inju-ries"[MeSH Terms] OR ("anterior"[All Fields] AND "cruciate"[All	All fields	42

	Fields] AND "ligament"[All Fields] AND "injuries"[All Fields]) OR "Anterior Cruciate Ligament Injuries"[All Fields] OR ("anterior"[All Fields] AND "cruciate"[All Fields] AND "ligament"[All Fields] AND "tear"[All Fields]) OR "anterior cruciate ligament tear"[All Fields]) OR ("anterior cruciate ligament reconstruction"[MeSH Terms] OR ("anterior"[All Fields] AND "cruciate"[All Fields] AND "ligament"[All Fields] AND "reconstruction"[All Fields]) OR "anterior cruciate ligament reconstruction"[All Fields]) OR ("ann clin lab res"[Journal] OR "aclr"[All Fields]) OR "Anterior Cruciate Ligament Injuries"[MeSH Terms] OR ("Anterior Cruciate Ligament Injuries"[MeSH Terms] OR ("anterior"[All Fields] AND "cruciate"[All Fields] AND "ligament"[All Fields] AND "injuries"[All Fields]) OR "Anterior Cruciate Ligament Injuries"[All Fields] OR ("anterior"[All Fields] AND "cruciate"[All Fields] AND "ligament"[All Fields] AND "rupture"[All Fields]) OR "anterior cruciate ligament rupture"[All Fields]))		
**Scopus**	(“Transcranial Neuromodulation” OR “Transcranial Direct Current Stimulation” OR tDCS OR “Transcranial Magnetic Stimulation” OR “Anodal Stimulation” OR “Cathodal Stimulation” OR “Transcranial Electrical Stimulation”) AND (“Anterior Cruciate Ligament” OR ACL OR “Anterior Cruciate Ligament Injur*” OR “Anterior Cruciate Ligament Tear” OR “Anterior cruciate ligament reconstruction” OR ACLR OR “Anterior Cruciate Ligament Rupture”)	Article title, abstract, keywords	45
**WOS**	(Transcranial Neuromodulation OR Transcranial Direct Current Stimulation OR tDCS OR Transcranial Magnetic Stimulation OR Anodal Stimulation OR Cathodal Stimulation OR Transcranial Electrical Stimulation) AND (Anterior Cruciate Ligament OR ACL OR Anterior Cruciate Ligament Injur* OR Anterior Cruciate Ligament Tear OR Anterior cruciate ligament reconstruction OR ACLR OR Anterior Cruciate Ligament Rupture)	All fields	61
**Cochran**	(Transcranial Neuromodulation OR Transcranial Direct Current Stimulation OR tDCS OR Transcranial Magnetic Stimulation OR Anodal Stimulation OR Cathodal Stimulation OR Transcranial Electrical Stimulation) AND (Anterior Cruciate Ligament OR ACL OR Anterior Cruciate Ligament Injur* OR Anterior Cruciate Ligament Tear OR Anterior cruciate ligament reconstruction OR ACLR OR Anterior Cruciate Ligament Rupture)	Title, abstract, keywords	20
**Total**			**168**
